# Association of miR‐1247‐5p expression with clinicopathological parameters and prognosis in breast cancer

**DOI:** 10.1111/iep.12287

**Published:** 2018-09-03

**Authors:** Peng Zhang, Changsheng Fan, Jun Du, Xueli Mo, Qikang Zhao

**Affiliations:** ^1^ Department of Breast Disease Peking University Shougang Hospital Beijing China

**Keywords:** breast cancer, clinicopathological parameters, miR‐1247‐5p, prognosis

## Abstract

Our study aimed to clarify the correlation between miR‐1247‐5p expression and clinicopathological parameters and survival of patients with breast cancer (BC). We evaluated the expression level of miR‐1247‐5p in 224 formalin‐fixed, paraffin‐embedded specimens (112 BC and matched cancer free tissues) by quantitative real‐time reverse transcriptase polymerase chain reaction (qRT‐PCR). miR‐1247‐5p expression in BC tissues was found to be decreased compared with matched normal tissues (*P* < 0.01). Additionally, low miR‐1247‐5p expression in BC tissues was significantly associated with the advanced TNM stage (*P* = 0.007), lymph node metastasis (*P* = 0.015), poorer pathological differentiation (*P* = 0.005) and molecular subtype (*P* = 0.027). The patients in the low miR‐1247‐5p group had a shorter disease‐free survival and overall survival than those in the high miR‐1247‐5p group (*P* < 0.01). Furthermore, the univariate and the multivariate analyses showed that miR‐1247‐5p expression was an independent predictor of overall survival (*P* < 0.01). Our study showed that miR‐1247‐5p was related to the biological behaviour of breast tumour and prognosis of patients with BC. miR‐1247‐5p could be a novel tumour suppressor and act as a potential biomarker and therapeutic agent for breast carcinoma.

## 1 INTRODUCTION

Currently, cancer is still one of the most common life‐threatening diseases worldwide. Breast cancer (BC) is the most common form of cancer and the second most common cause of cancer death in women.^1^ In recent years, BC incidence rates are increasing in the majority of countries. But in contrast, BC death rates have declined as a result of improved treatment and early detection through mammography.^2^ Although great advances have been made in clinical treatments, satisfactory outcomes have not always been achieved.^3^ Thus prevention and treatment of BC still remains a concern and challenge for oncologists throughout the world.^4^, ^5^ Early detection is one of the most important methods which might the survival of BC patients, so it remains significant to find new biomarkers for diagnosis and prognosis estimation of BC patients.

MicroRNAs are small noncoding RNA molecules that function in RNA silencing and post‐transcriptional regulation of gene expression.^6^ A variety of microRNAs have links with cancer and affect cancer development.^7^ Many reports have shown that microRNAs are associated with cancer metastasis and prognosis.^8^, ^9^, ^10^ More and more studies showed that microRNAs could be promising biomarkers for tumour diagnosis, prediction of therapy response and prognosis.^11^, ^12^, ^13^

As a member of the miR‐1247 family, miR‐1247‐5p plays a critical role in tumour progression. Generally, its expression has been reported to decrease in human cancers. More and more data show that miR‐1247‐5p, a novel tumour suppressor, could act as a potential biomarker and therapeutic agent for a variety of cancers.^14^, ^15^, ^16^ However, correlative studies on BC have not been reported. In this study, we investigated the expression levels of miR‐1247‐5p in BC tissues and analysed the association of its expression with clinicopathological factors and clinical prognosis.

## 2 MATERIALS AND METHODS

### 2.1 Specimens

We collected 224 formalin‐fixed, paraffin‐embedded (FFPE) samples for our study. All specimens, including 112 BC tissues and paired adjacent normal tissues, were obtained from 112 patients who were treated with surgery at Peking University Shougang Hospital between January 2005 and December 2009. The collected criteria of adjacent normal tissues was 10 mm minimum distance from the tumour tissues. The diagnosis and classification of BC patients were based on the tumour‐node‐metastasis (TNM) system of American Joint Committee on Cancer (AJCC).^17^ After resection, all paraffin‐embedded tissue specimens were cut into slices and were stained with haematoxylin and eosin as control for selecting aimed samples. We chose those sections that contain more than 90% cancer cells for total RNA extraction and subsequent analyses.

### 2.2 Ethical approval

This investigation was approved by the Medical Ethics Committee of Peking University Shougang Hospital. Each patient signed their own consent form.

### 2.3 RNA extraction and reverse transcription

Total RNA extraction from tissues was conducted using the miRNeasy FFPE Kit (Qiagen, Hilden, Germany) according to the instruction offered by the company. The reverse transcription was performed using TaqMan MicroRNA Reverse Transcription Kit (Haoqin Biotechnology, Shanghai, China). The miR‐1247‐5p‐specific primer and U6 small nuclear RNA (as an internal reference) primer were obtained from the TaqMan MicroRNA Assays. Cycling condition was 1 cycle at 95°C for 10 minute, 40 cycles at 95°C for 15 second, 57°C for 30 second and 72°C for 30 second. For this study each RT‐PCR experiment was performed three times. Relative quantification (RQ) refers to the relative expression level of miR‐1247‐5p to U6 (internal reference gene). For every sample the value of RQ was calculated using the 2^−ΔCt^ method, where ΔCt = Ct_miR‐1247‐5p_‐Ct_U6_.^18^

### 2.4 Statistical analysis

The expression difference of miR‐1247‐5p in BC and in normal tissues was analysed using the Mann‐Whitney test. Measurement data were analysed by Student's *t* test, and X^2^ test was used to analyse the categorical data. Survival rates were evaluated using the Kaplan‐Meier method, and Gehan‐Breslow‐Wilcoxon test was used to assess the differences of survival rates in different groups. A Cox regression model was applied for univariate and multivariate analyses. All data were analysed by spss version 17.0 (SPSS, Chicago, IL, USA). In this study, *P *<* *0.05 was considered statistically significant.

## 3 RESULTS

### 3.1 Expression levels of miR‐1247‐5p decreased in BC tissues

**Figure 1:**
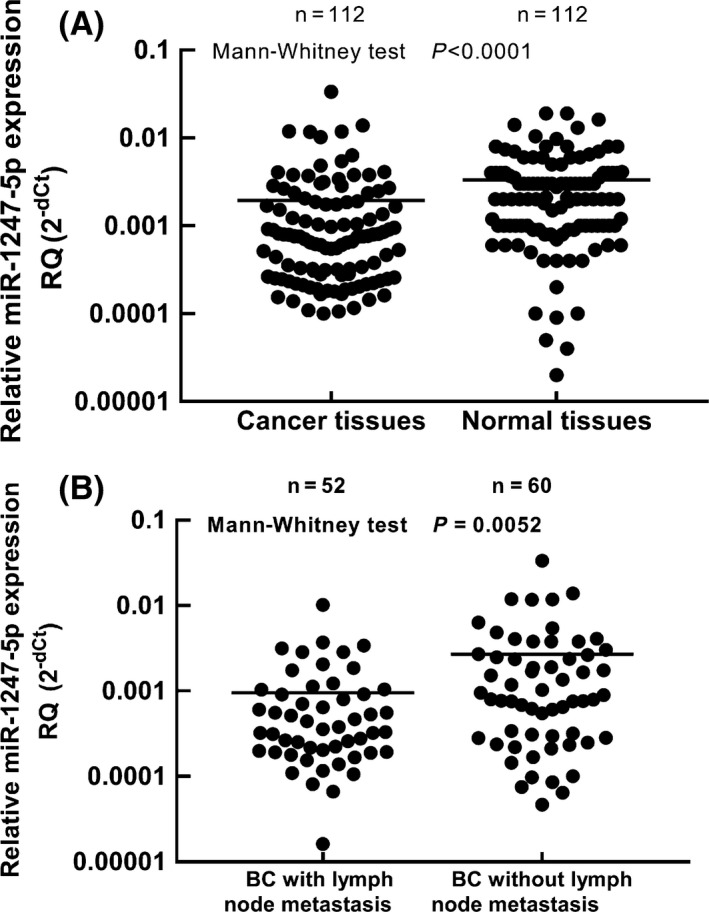
Expression levels of miR‐1247‐5p in breast cancer (BC) and noncancer tissues. RQ refers to the relative expression level of miR‐1247‐5p to U6 (internal reference gene). A, Comparison of miR‐1247‐5p expression levels in BC and noncancer tissues in 112 matched specimens. The expression levels of miR‐1247‐5p were higher in noncancer tissues than in BC tissues (*P *<* *0.0001); B, Comparison of miR‐1247‐5p expression levels in BC (BC) tissues with and without lymph node metastasis. The expression levels of miR‐1247‐5p were significantly lower in BC with lymph node metastasis than those without lymph node metastasis (*P *=* *0.0052)

We tested the expression levels of miR‐1247‐5p in 112 BC tissues and matched adjacent nontumour tissues. As shown in Figure 1A, compared with adjacent normal tissues, BC tissues showed lower expression levels of miR‐1247‐5p (*P *<* *0.0001). To evaluate the relation between miR‐1247‐5p expression and early‐stage metastasis of BC, we compared and analysed the expression levels of miR‐1247‐5p in BC with and without lymph node metastasis. As shown in Figure 1B, the expression levels of miR‐1247‐5p in BC tissues with lymph node metastasis (mean: 0.000893) decreased to 33.7% of those without lymph node metastasis (mean: 0.002649) (*P = *0.0052). These data suggested that miR‐1247‐5p might function as a tumour suppressor to prevent progression of BC.

### 3.2 Low expression levels of miR‐1247‐5p correlated with advanced TNM stage, lymph node metastasis and poorer pathological differentiation

To further evaluate whether low expression of miR‐1247‐5p was related to the clinical progression of BC, we classified its expression levels as low and high level groups according to the median value and then analysed the relation between miR‐1247‐5p expression and the clinicopathological features of BC patients. As summarized in Table 1, low expression of miR‐1247‐5p was associated significantly with advanced TNM stage (*P *=* *0.007), lymph node metastasis (*P *=* *0.015), poorer pathological differentiation (*P *=* *0.005) and molecular subtype (*P *=* *0.027). No significant associations were found between miR‐1247‐5p expression level and patients’ age, tumour size or venous invasion (*P *>* *0.05). These data showed that downregulation of miR‐1247‐5p was associated with malignant biological behaviour of BC.

**Table 1:** Association of miR‐1247‐5p expression and clinicopathological parameters of breast cancer

Variable	Category	No.	miR‐1247‐5p expression		*P*
			Low	High	
Age (years)	<50	39	21	18	0.552
	≥50	73	35	38	
Tumour size (cm)	<3	53	27	26	0.850
	≥3	59	29	30	
Lymph node metastasis	Absent	60	22	38	0.015^a^
	Present	52	31	21	
TNM stage	I/II	71	28	43	0.007^b^
	III	41	27	14	
Degree of differentiation	Well and moderate	74	25	49	0.005^b^
	Poor	38	24	14	
Venous invasion	Negative	75	36	39	0.547
	Positive	37	20	17	
Molecular subtype	Luminal A/B	72	27	45	0.027^a^
	Her‐2/basal‐like	40	25	15	

^a^ *P *< 0.05; ^b^ *P *< 0.01.

### 3.3 Downregulation of miR‐1247‐5p correlated with poor survival of BC patients

**Figure 2:**
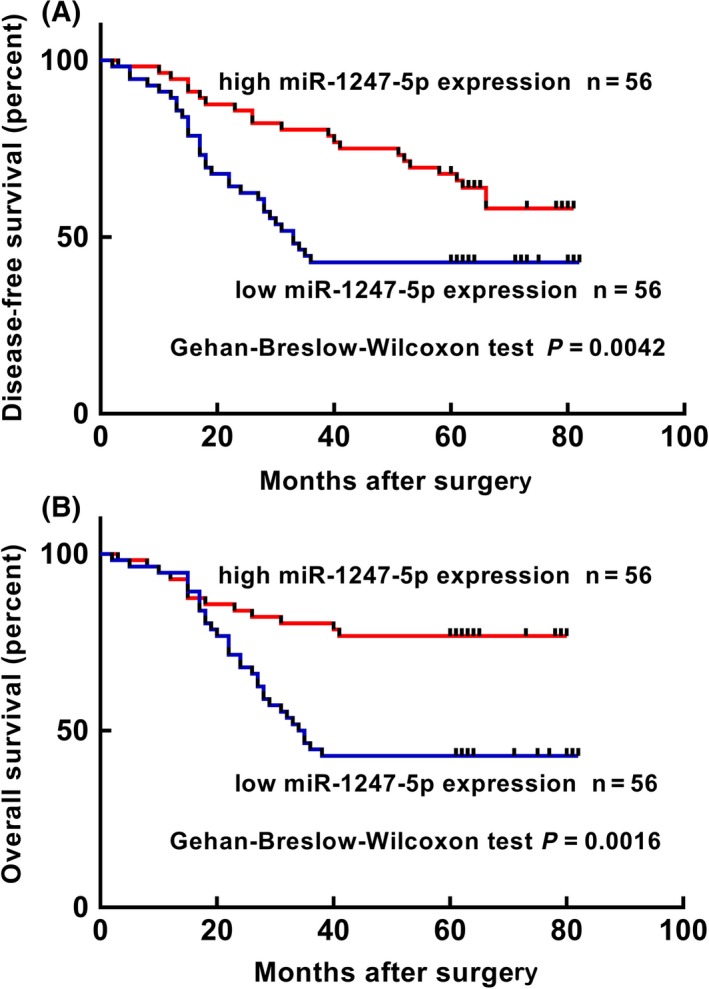
Survival curves of patients with breast cancer (BC) based on the basis of miR‐1247‐5p expression levels. A, Kaplan‐Meier disease‐free survival (DFS) curves of patients with BC (BC) on the basis of miR‐1247‐5p expression levels. Patients of the low level group had significantly lower DFS rates than those of the high level group (Gehan‐Breslow‐Wilcoxon test, *P *=* *0.0042); B, Kaplan‐Meier overall survival curves (OS) of patients with BC on the basis of miR‐1247‐5p expression levels. Patients of the low expression group had significantly lower OS rates than those in the high level group (Gehan‐Breslow‐Wilcoxon, *P *=* *0.0016) [Colour figure can be viewed at http://wileyonlinelibrary.com]

Kaplan‐Meier analyses were performed to investigate the association between miR‐1247‐5p expression and the survival of BC patients. We plotted disease‐free survival (DFS) and overall survival (OS) curves as shown in Figure 2. The results showed that the patients with low miR‐1247‐5p expression exhibited poorer DFS rates (*P *=* *0.0042) and OS rates (*P *=* *0.0016) than those with high miR‐1247‐5p expression.

The univariate and the multivariate analyses were also performed to identify factors related to patient prognosis. As shown in Table 2, the univariate Cox proportional hazard regression analysis revealed that TNM stage, lymph node metastasis, venous invasion and miR‐1247‐5p expression levels were predictive factors for prognosis of BC patients (*P *<* *0.05). Furthermore, a multivariate analysis showed that expression level of miR‐1247‐5p was an independent prognostic factor of overall survival in patients with BC (*P *=* *0.007) (Table 3). These data indicated that the downregulation of miR‐1247‐5p was associated with poor prognosis of BC patients.

**Table 2:** Univariate analysis of clinicopathological parameters for overall survival of breast cancer patients

Variable	Cases	HR	95%CI	*P* value
Age (years)			0.711‐2.370	0.395
<50	39	1		
≥50	73	1.298		
Tumour size (cm)			0.935‐3.208	0.081
<3	53	1		
≥3	59	1.732		
Lymph node metastasis			3.749‐17.692	0.000^b^
Absent	60	1		
Present	52	8.144		
TNM stage			2.327‐8.627	0.000^b^
I/II	71	1		
III	41	4.481		
Degree of differentiation			0.920‐3.377	0.087
Well and moderate	74	1		
Poor	38	1.763		
Venous invasion			2.211‐8.451	0.000^b^
Negative	75	1		
Positive	37	4.323		
Molecular subtype			1.879‐3.378	0.015
Luminal A/B	72	1		
Her‐2/basal‐like	40	2.025		
miR‐1247‐5p expression			1.366‐4.802	0.003^b^
High	56	1		
Low	56	2.561		

^a^ *p*<0.05; ^b^ *p<*0.01.

**Table 3:** Multivariate analysis of clinicopathological parameters for overall survival of breast cancer patients

Variable	Cases	HR	95%CI	*P* value
TNM stage			1.650‐13.814	0.004^b^
I/II	71	1		
III	41	4.775		
Lymph node metastasis			7.903‐98.215	0.000^b^
Absent	60	1		
Present	52	27.860		
Venous invasion			1.165‐4.872	0.017^a^
Negative	75	1		
Positive	37	2.382		
miR‐1247‐5p expression			1.289‐4.817	0.007^b^
High	56	1		
Low	56	2.491		

^a^ *p*<0.05; ^b^ *p<*0.01.

## 4 DISCUSSION

Recently, more and more studies have indicated that microRNAs are associated with tumorigenesis and development^19^, ^20^, ^21^ and that microRNAs play important roles in a variety of biological processes; their dysregulation may be crucial to cancer initiation, progression and treatment outcome.^22^, ^23^ Some microRNAs were also reported to be associated with BC. In recent study, seven microRNAs (miR‐21, miR‐96, miR‐141, miR‐182, miR‐183, miR‐200a and miR‐429) were upregulated and two microRNAs (miR‐139 and miR‐145) were downregulated in human BC.^24^ A systematic review showed that two candidate microRNAs (miR‐21 and miR‐210) were upregulated and six microRNAs (miR‐99a, miR‐139‐5p, miR‐145, miR‐195, miR‐205 and miR‐497) were downregulated in BC tissues comparing with normal tissues.^25^ Many research have showed that miR‐1247‐5p was downregulated in cancer tissues, and it act as a tumour inhibition factor.^14^, ^15^, ^16^, ^26^, ^27^, ^28^ In accord with the above results, our study suggested that miR‐1247‐5p was downregulated in BC tissues relative to cancer free tissues, indicating that downregulation of miR‐1247‐5p expression was associated with carcinogenesis and cancer development of BC.

As comparison, a few reports have shown upregulation of miR‐1247‐5p expression in cancer tissues or blood of cancer patients.^29^, ^30^ The discordance may be explained by the discrepancy of molecular regulation mechanism in different types of tumour tissues. Additionally, Wang et al reported that miR‐1247‐5p was upregulated in the pancreatic juice from pancreatic ductal adenocarcinoma patients compared with which from noncancer patients.^30^ The inconsistent results were probably due to the different tumour microenvironment(s) which act on tumour cells and affected the expression of miR‐1247‐5p. The latest studies showed that microRNAs have interaction with the tumour microenvironment *via* a variety of molecular pathways.^31^, ^32^, ^33^ Shi et al investigated the expression profile of miR‐1247‐5p in pancreatic cancer tissue microarray by in situ hybridization and found that it was significantly downregulated in pancreatic cancer tissues compared to matched benign tissues.^14^ The different expression pattern in pancreatic cancer tissues and in pancreatic juice from pancreatic cancer patients may be explained by above tumour microenvironment influence. miR‐1247‐5p show different expression levels in different tissues because of complicated microenvironment factors’ roles.

We analysed the association of miR‐1247‐5p expression with clinicopathological parameters and patients survival in BC. The results showed that miR‐1247‐5p dysregulation correlated with TNM stage and lymph node metastasis, indicating that miR‐1247‐5p is involved in the tumorigenesis and progression of BC. Furthermore, we found that lower miR‐1247‐5p expression was significantly associated with decreased DFS and OS of patients with BC. On the contrary, higher miR‐1247‐5p expression correlated with better DFS and OS, suggesting that miR‐1247‐5p may be used as a prognostic biomarker in BC. Additionally, we assessed several clinicopathological parameters of BC patients using Cox regression. We drew the conclusion that miR‐1247‐5p was an independent prognostic factor, and it might be used as a promising biomarker of early metastasis and prognosis for patients with BC.

Regulation of signalling pathway of miR‐1247‐5p involvement in initiation, progression and metastasis of BC has not been clarified. It was reported that miR‐1247‐5p expression correlated with prognosis of pancreatic cancer and it inhibits cell proliferation by targeting neuropilin1 (NRP1) and neuropilin2 (NRP2). Increased expression of miR‐1247‐5p inhibited proliferation, tumorigenicity, colony formation and triggered G0/G1 cell cycle arrest in pancreatic cancer cells.^14^ Zhao et al reported that the significant downregulated miR‐1247‐5p was a potential tumour suppressor by targeting MAP3K when they investigated microRNA expression profiles in stem cells of osteosarcoma (OS).^15^ Several molecules involved in cell proliferation (*FGFR4*), migration (*BAIAP2L1* and *PTK2*), apoptosis (*FAM129B*) and a thyroid‐specific transcription factor (*PAX8*) were found to be involved in regulation of microRNAs when miR‐1247‐5p was less expressed in all tumours with a follicular pattern of growth (follicular adenomas, follicular carcinomas and follicular variant of papillary tumours) than in normal tissues.^27^ Scaravilli et al found that MYCBP2 (myc‐binding protein 2) was the target gene of miR‐1247‐5p when they investigated the association between microRNAs and the tumour biological behaviour of castration‐resistant prostate cancer. Recently, Juan Zhang et al^16^ reported that overexpression of miR‐1247‐5p dramatically inhibited non‐small‐cell lung cancer cell growth, migration, invasion and cell cycle progression and Stathmin 1 (STMN1) was found to be an immediate and functional target of miR‐1247‐5p.

Our study was the first one about the relationship between miR‐1247‐5p expression and BC. The regulation mechanism of miR‐1247‐5p and its target genes will be investigated in our future study.

Thus in this study we confirmed that miR‐1247‐5p expression was significantly downregulated in BC tissues. Additionally, downregulation of miR‐1247‐5p was associated with TNM stage, lymph node metastasis and poor survival of patients with BC. We also demonstrated that miR‐1247‐5p was an independent outcome predictor. Our conclusion was that miR‐1247‐5p is a potential biomarker of early metastasis diagnosis and prognosis estimation, and could be a promising therapeutic index.
